# Head-to-Head Comparison of ^68^Ga-PSMA-11 and ^131^I in the Follow-Up of Well-Differentiated Metastatic Thyroid Cancer: A New Potential Theragnostic Agent

**DOI:** 10.3389/fendo.2021.794759

**Published:** 2021-12-22

**Authors:** Quetzali Pitalua-Cortes, Francisco Osvaldo García-Perez, Joel Vargas-Ahumada, Sofia Gonzalez-Rueda, Edgar Gomez-Argumosa, Eleazar Ignacio-Alvarez, Irma Soldevilla-Gallardo, Liliana Torres-Agredo

**Affiliations:** ^1^ Nuclear Medicine and Molecular Imaging Department, National Cancer Institute, Mexico City, Mexico; ^2^ Nuclear Medicine Department, Universidad Autónoma de Bucaramanga, Bucaramanga, Colombia

**Keywords:** thyroid cancer, PSMA, iodine, theragnostic, PET

## Abstract

**Introduction:**

Thyroid cancer is the main endocrine neoplasia worldwide, for which ^131^I therapy is the cornerstone treatment. One of the main problems of follow up in patients with this type of cancer, is the need for thyroglobulin stimulation, not to mention the poor availability of ^123^I or ^124^I, to perform studies with a higher degree of sensitivity. Prostatic Specific Membrane Antigen (PSMA) PET/CT has demonstrated to be quite useful in a diversified number of neoplasms, on behalf of its capacity of evaluating the extent of type II carboxypeptidase expression in vascular endothelium. The end point of this article is to assess whether this novel image method possesses applicability in thyroid neoplasms follow up, for diagnostic and potentially therapeutic purposes.

**Methods:**

We retrospectively evaluated well differentiated metastatic thyroid cancer patients, who underwent a post therapeutic ^131^I dose whole body scan (WBS) and complementary SPECT/CT, as well as ^68^Ga-PSMA–11 PET/CT.

**Results:**

Ten patients with differentiated thyroid cancer were included, of whom 80% were women and 20% men, mean age was 58 years old (± 11.6). Sixty-four metastatic lesions were analyzed, 67.19% had papillary histology and 32.81% were follicular type, the most affected site of metastases was bone in 57.81%, followed by lung 17.19%, lymph nodes 7.81%, postoperative thyroid bed 4.69%, brain 4.69% and others 7.81%. ^68^Ga PSMA-11 PET/CT detected 64/64 lesions, all of them also identified by computed tomography (CT), whereas ^131^I SPECT/CT detected 55/64 lesions. Discrepant lesions were localized in lung 44.4%, brain 22.2%, postoperative thyroid bed 11.1%, lymph nodes 11.1% and bone 11.1%. The degree of correspondence among observers was outstanding for both radiotracers, but close upon perfect for PSMA-11 (κ = 0.98; 95% CI, 0.80 – 0.91), as opposed to ^131^ I (κ = 0.86; 95% CI, 0.71 – 0.76).

**Conclusions:**

^68^Ga-PSMA PET/CT showed an utterly superior capability for metastatic lesion detection when compared to ^131^I SPECT/CT. These findings suggest that PSMA PET/CT could possibly and precociously identify radioiodine refractoriness. PSMA uptake values not only expedite diagnosis, but also award it the ability to be used for therapeutic intents.

## Introduction

Thyroid cancer is the most common endocrine malignancy, with approximately 62,000 new cases per year in the USA (1). Differentiated thyroid cancer (DTC) is a slow growing tumor with a very low disease-specific mortality rate for local-regional disease (5 – year overall survival of 99.9% and 98.3% for localized and regional metastatic disease respectively), however distant metastatic disease is associated with significantly worse prognosis (5 – year overall survival of 54.9%) (1). It is estimated that approximately 2,200 cancer deaths will occur among patients with thyroid carcinoma in the United States (2). Oncological outcomes in thyroid cancer depend on histologic subtypes, age, gender, tumor characteristics, molecular features, and lymph node or distant metastases. Most thyroid cancer patients have a favorable response to surgery and risk-adapted postoperative therapy with thyroid hormone suppression and radioactive iodine (RAI) therapy, in selected situations (3).

Standard of care imaging modalities include diagnostic ^131^I – WBS after surgery to assess the completeness of thyroidectomy and whether residual disease is present. Post-treatment ^131^I whole body imaging must be done, considering that up to 25% of scans exhibit lesions that may be clinically important, which were not detected by other diagnostic imaging methods (4). However, some of the inherent limitations to this method, include patient preparation (mainly levothyroxine withdrawal, which decreases quality of life due to the exacerbation of hypothyroidism symptoms, among others) and low diagnostic accuracy (planar scan has a 41% sensitivity and 68% specificity, while sensitivity of SPECT reaches up to 45% with an 89% specificity) (5).

DTC can become resistant to RAI therapy, and in those cases, treatment options are limited (6). ^18^F-FDG PET/CT scan is indicated for patients with a negative ^131^I WBS, who have suspicion of structural disease based on other imaging methods and/or elevated thyroglobulin (Tg) suggestive of distant metastases (7). Thus, imaging and therapy in thyroid cancer would largely be benefited from improvement.

Novel molecular radiotracers utilizing PSMA ligand uptake measured with PET/CT have emerged as a new generation imaging modality. PSMA is a type II transmembrane glycoprotein highly expressed in prostate cancer (PCa) and is the upcoming imaging modality for staging, re-staging and response assessment in PCa (8). It has showed better diagnostic accuracy than conventional imaging in high-risk PCa patients, and therapeutic benefits (with safety and efficacy) when combined with ^177^Lu (9, 10). Despite its name, it is not specific to prostate; PSMA has been found to be expressed in the neovasculature of multiple solid tumors, and increased uptake of ^68^Ga-PSMA PET/CT has been demonstrated in several non-prostatic malignancies, including thyroid cancer (11, 12). Specifically, histological studies have verified the expression of PSMA in the microvasculature of thyroid cancers (13, 14) where, PSMA expression was related to malignant disease, poor prognostic factors and poorer progression free survival (PFS) (15). This evidence suggests the potential use of PSMA PET/CT as a theragnostic and prognostic imaging biomarker. Given this clinical and technical background, we report our experience in a head-to-head comparison of these two imaging procedures for the detection of disease in patients affected by thyroid cancer.

## Materials and Methods

### Study Population

This retrospective study was approved by our hospital’s Local Research Ethics Committee; due to its retrospective condition, informed consent from patients was not necessary.

This study incorporates patients that were referred to the nuclear medicine department in the period comprehended between 2019 – 2020, with the following inclusion criteria: (1) patients with histologically proven well differentiated thyroid cancer, (2) who had received radioiodine therapy, and (3) no other anti-tumor therapy prior to PET/CT. Exclusion criteria were patients (1) who had other primary malignancies at the time of examination, (2) with prior history of neck radiotherapy. FDG PET/CT was not performed on any patient, since we were not looking for tumoral dedifferentiation.

Every patient that received an ^131^I ablative or therapeutic dose, was prepared according to ATA guidelines, verifying TSH levels (above 30 µUI/L) prior to the radiopharmaceutical administration. None of them received rhTSH. 

### Image Acquisition

WBS and SPECT/CT from vertex to pelvis (Siemens SPECT/CT Symbia T6, Siemens Healthineers, Knoxville, TN, US) were performed 7 days after therapeutic oral administration of ^131^ I (3,700 to 7,400 MBq), according to the clinical standard protocol. Regarding the timing protocol for this study, we followed ATA guidelines recommendations of performing WBS in a period ranging from 2 to 12 days post therapy, considering the dose of ^131^I given, and this radiotracer’s half-life. The SPECT reconstruction datasets were: 128 × 128 matrix (ordered subset expectation maximization [OSEM]) algorithms with 8 subsets and 4 iterations, followed by an 8 – mm Gaussian filter.

Whole – body (from top of head to mid – thigh) PET/CT was performed after 6 weeks from SPECT/CT acquisition, approximately 60 minutes after the intravenous injection of ^68^Ga PSMA–11 (148 – 185 MBq) according to the clinical standard protocol for tumor imaging. A Biograph mCT 20 Excel PET/CT scanner was used (Siemens Healthineers, Knoxville, TN, US). The PET reconstruction datasets were 400 × 400 matrix (pixel size: 1.5625 × 1.5625 × 2.78 mm^3^) with Time of Flight (TOF) OSEM algorithms with 21 subsets and 3 iterations, followed by a 6 – mm Gaussian filter. CT was acquired using 140 mA, 130 kV, 5 mm width and a 1 mm pitch.

### Image Analysis

Two experienced nuclear physicians evaluated the resulting images in consensus. Both studies of each patient were compared, identifying the areas of greatest radiotracer uptake, and associating them with corresponding CT findings. Lesions were classified by regions in lymph nodes, postoperative thyroid bed, bone, brain, lung, and others (muscle and kidneys). Quantified lung lesions were larger than 1 cm, a maximum of 4 lesions.

Regarding SPECT/CT, counts of each lesion were quantified, as well as those from background (pectoralis major muscle) to obtain tumor–to–background ratios (TBR = lesion counts/background counts). As for PET/CT, the SUVmax was measured with isocontour volume of interest (VOI), along with the determination of the background with a spherical VOI of 1 cm^3^ in the pectoralis major muscle, which was used as a reference to calculate the TBR (TBR = SUVmax lesion/SUVmax tissue reference).

### Reference Standard

Lesions that went through biopsy, or that presented tomographic alterations associated with uptake of at least one of the radiopharmaceuticals, were taken as true positives. Positive lesions in PET and SPECT were considered those with TBR > 1.

### Statistical Analysis

Collected data was analyzed using the statistical software STATA 14.0. The univariate analysis carried out included the clinical characterization of the patients. Descriptive statistic was performed, providing frequencies for categorical variables; for continuous variables, the standard deviation (SD), mean and median were provided.

The sample size was determined by convenience, due to the retrospective characteristic.

## Results

We evaluated 10 patients with differentiated thyroid cancer: 80% (n = 8) were women and 20% (n = 2) men, mean age was 58 years old (± 11.6). The characteristics of the population, including histology, are summarized in **Table 1**.

**Table 1:** Patient characteristics.

Patient	Age (years)	Gender	Histopatology	Thyroglobulin (ng/mL)	Anti- Thyroglobulin antibodies (U/ML)	Radioiodine cumulative dose (MBq)	Treatment received	Metastatic lesion localization	RAI lesion uptake (TBR)	PSMA lesion uptake (TBR)
**1**	64	Male	Papillary thyroid carcinoma, classic variant	264.5	4000	7400	Total Thyroidectomy (TT) + Radioactive iodine (RAI) therapy	Lymph nodes, thyroid bed, lung and bone.	0 – 714.2	3 – 14.9
**2**	42	Male	Papillary thyroid carcinoma, classic variant	114	12	16650	TT + RAI	Lymph nodes.	0	1.8
**3**	52	Female	Papillary thyroid carcinoma, classic variant	1000	17.7	7400	TT + RAI	Brain, bone, muscle.	0 – 544	1.4 – 35.2
**4**	73	Female	Papillary thyroid carcinoma, classic variant	546	2.3	14800	TT + RAI	Lung and retrotracheal implant.	0 – 7.5	3.3 – 6.5
**5**	61	Female	Follicular thyroid carcinoma, insular differentiation (20%)	3922	1.55	25900	TT + RAI	Bone and paravertebral implant.	1.9 – 10.3	6 – 11.3
**6**	53	Female	Follicular thyroid carcinoma, angioinvasive with oxyphilic switch	1444	1.2	33300	TT + RAI	Bone.	3 – 10.5	2.5 – 4.9
**7**	65	Female	Papillary thyroid carcinoma, classic variant	253	2	14800	TT + RAI	Thyroid bed and lymph nodes.	3 – 12.6	3.3 – 5.6
**8**	34	Female	Papillary thyroid carcinoma, with solid areas (10%) and hobnail micropapillary (5%)	219	1	29600	TT + RAI	Thyroid bed and lung.	1	3 – 5
**9**	55	Female	Papillary thyroid carcinoma, classic variant	1384	10.5	22200	TT + RAI	Lymph nodes, bone, kidneys.	0 – 13	2.2 – 17
**10**	63	Female	Follicular variant of Papillary thyroid carcinoma, tall cell variant (10%), insular variant (20%)	2046	174.6	46250	TT + RAI	Lymph nodes, lung and brain.	1.6 – 14.8	8 – 74

Sixty-four metastatic lesions were analyzed, 67.19% (n = 43) had papillary histology and 32.81% (n = 21) were follicular type, the most affected site of metastases was bone in 57.81% (n = 37), followed by lung 17.19% (n = 11), lymph nodes 7.81% (n = 5), postoperative thyroid bed 4.69% (n = 3), brain 4.69% (n = 3) and others 7.81% (n = 5).

**Figure 1:**
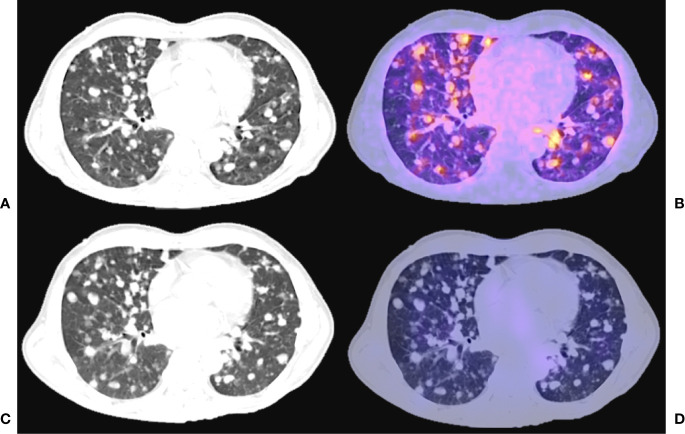
55 years old female, papillary thyroid carcinoma classic variant, treated with thyroidectomy + RAI (22200 MBq cumulative dose). Thyroglobulin 1384 ng/ml. Fused hybrid and simple CT thoracic images. **(A)** CT acquired for PET fusion **(B)** ^68^ Ga - PSMA PET/CT. **(C)** CT acquired for SPECT fusion **(D)** ^131^I -SPECT/CT.

**Figure 2:**
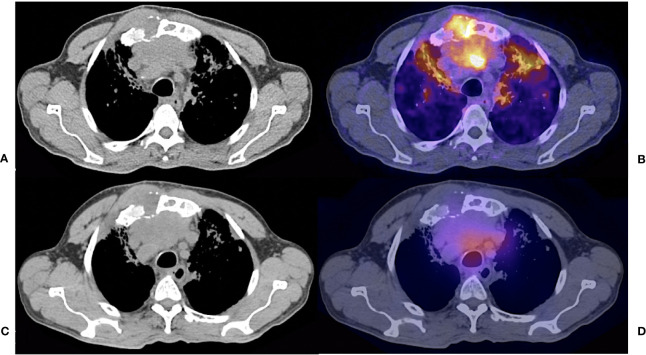
61 years old female with follicular thyroid carcinoma, insular differentiation (20%), treated with TT + RAI (cumulative dose 25900 MBq); Thyroglobulin 3922 ng/dl. Fused hybrid and simple CT thoracic images shows a Iytic lesion in sternum with soft tissue component and prevascular conglomerate. **(A)** CT acquired for PET fusion **(B)** ^68^Ga – PSMA PET/CT with focal uptake of the radiotracer. **(C)** CT acquired for SPECT fusion **(D)** ^131^I – SPECT/CT shows diffuse uptake of the radiotracer.

**Figure 3:**
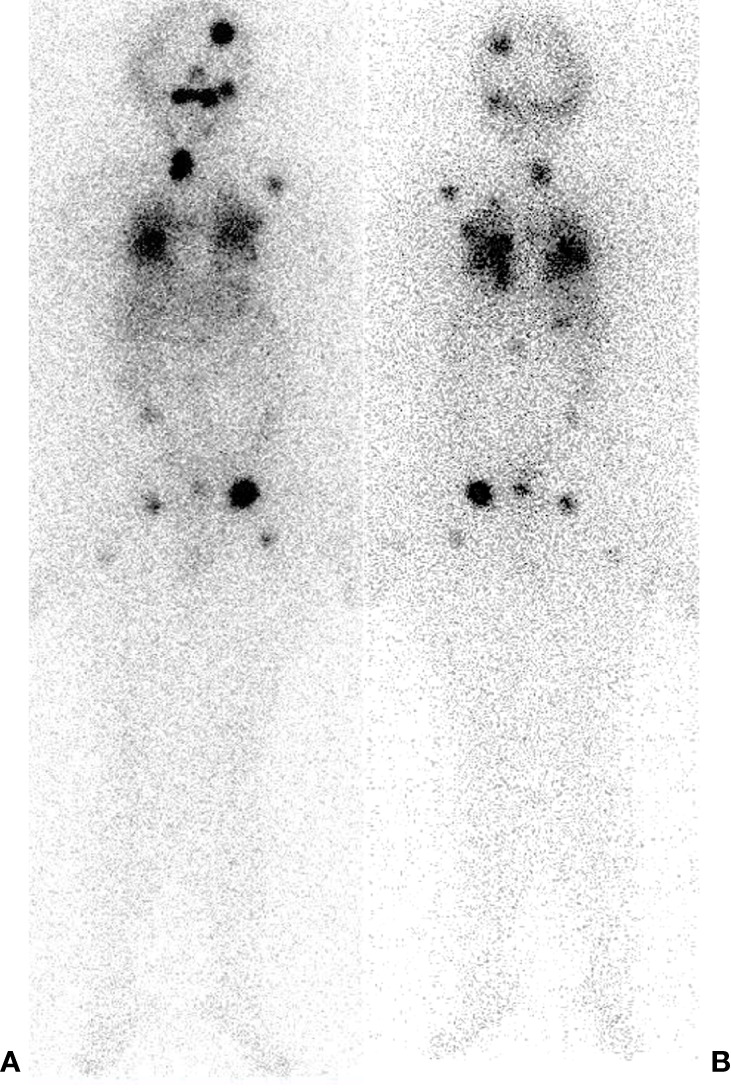
^131^I – Post Therapy Whole Body Scan. 42 years old male, papillary thyroid carcinoma classic variant. Thyroglobulin 114. RAI cumulative dose (16650 MBq). **(A)** Anterior projection. **(B)** Posterior projection.

**Figure 4:**
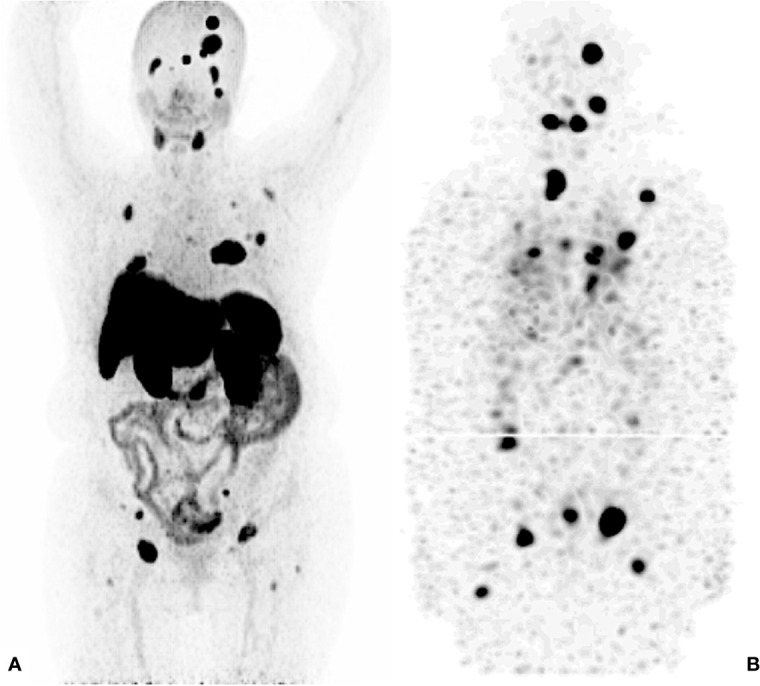
^131^I – Post Therapy Whole Body Scan. 42 years old male, papillary thyroid carcinoma classic variant. Thyroglobulin 114. RAI cumulative dose (16650 MBq) Maximum Intensity Projections. **(A)** ^68^ Ga - PSMA PET/CT. **(B)** ^131^I - SPECT/CT.

**Figure 5:**
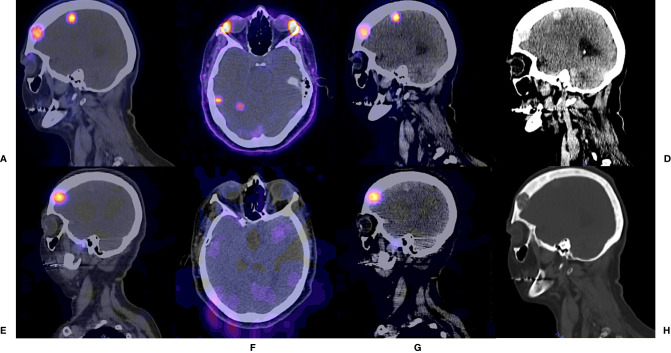
52 years female, papillary thyroid carcinoma classic variant. Thyroglobulin 1000; radioiodine cumulative dose 7400 MBq. Fused hybrid and simple CT head and neck images. **(A–C)** show ^68^ Ga – PSMA PET/CT sagittal **(A, C)** and axial **(B)** views in soft tissue **(A, B)** and brain windows **(C)**; while **(E–G)** demonstrate ^131^I – SPECT/CT images in homologous reconstructions. Notice how PSMA PET/CT illustrates more intraparenchymal metastatic lesions, which also show higher tracer uptake than ^131^—SPECT/CT. It also can be visualize a bone lytic lesión in frontal bone. Images on the right represent simple CT sagittal recontructions in brain **(D)** and bone **(H)** windows, where morphologic aspects of the lesions are better characterized.

**Figure 6:**
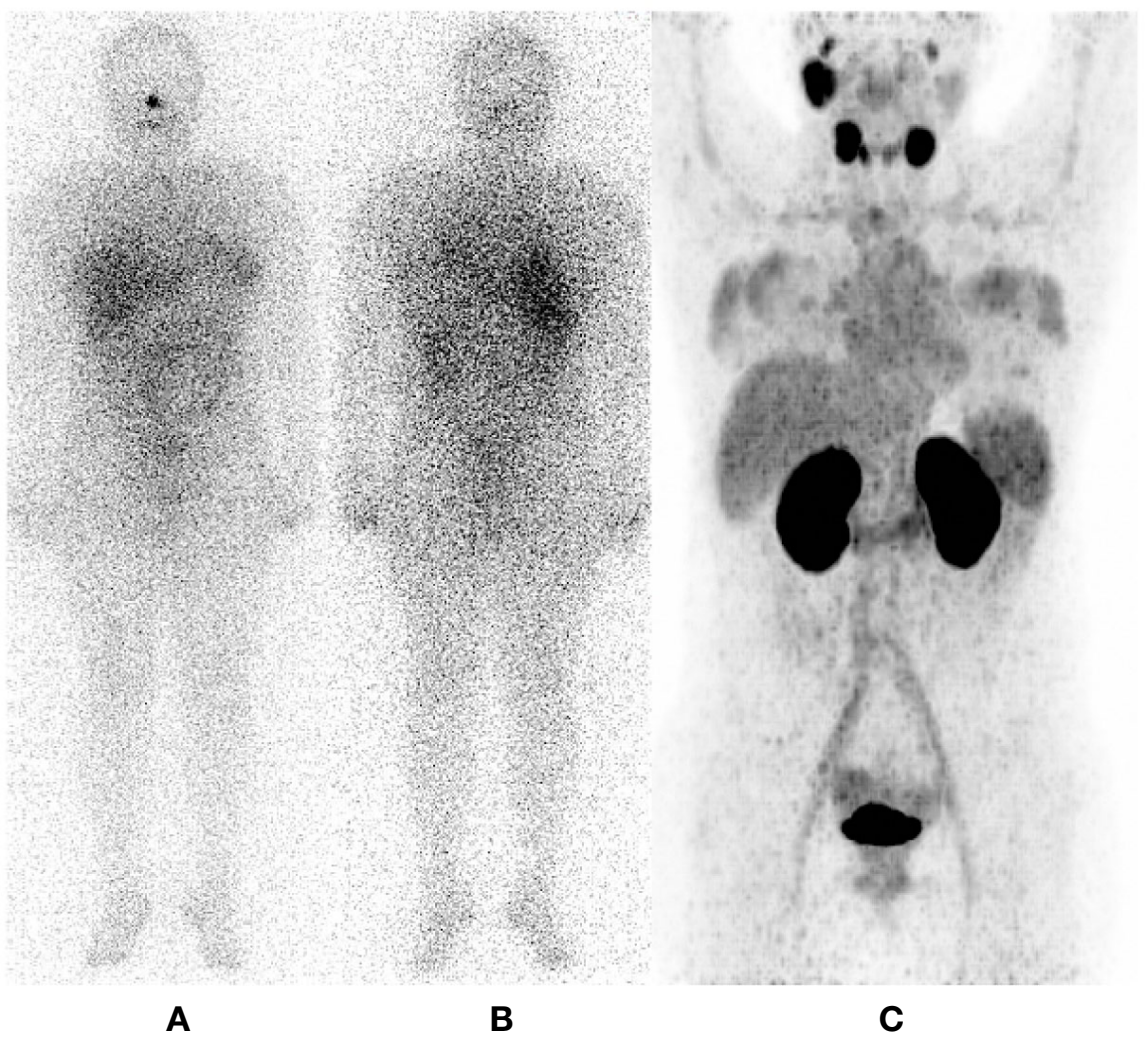
73 years old female treated with total thyroidectorny + RAI (cumulative dose 14800 MBq); Thyroglobulin 546 ng/ml. **(A, B)** ^121^I – post therapy whole body scan shows no uptake other than usual biodistribution. Physiological uptake variant was observed in mamary glands with both tracers. **(C)** ^68^Ga – PSMA PET/CT MIP, on the other hand, conspicuously shows a central cervical compartment adenopathy.

**Figure 7:**
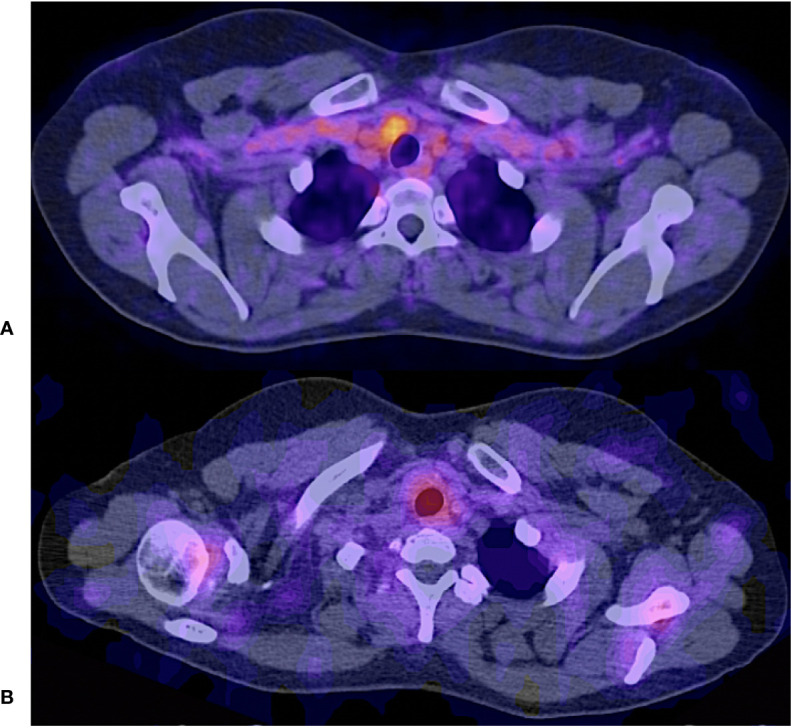
Fused hybrid images **(A)** ^68^Ga PSMA PET/CT shows focal radiotracer uptake in the cervical level VI located adenopathy. **(B)** ^131^I – SPECT/CT shows diffuse radiotracer uptake in the same adenopathy.

**Figure 8:**
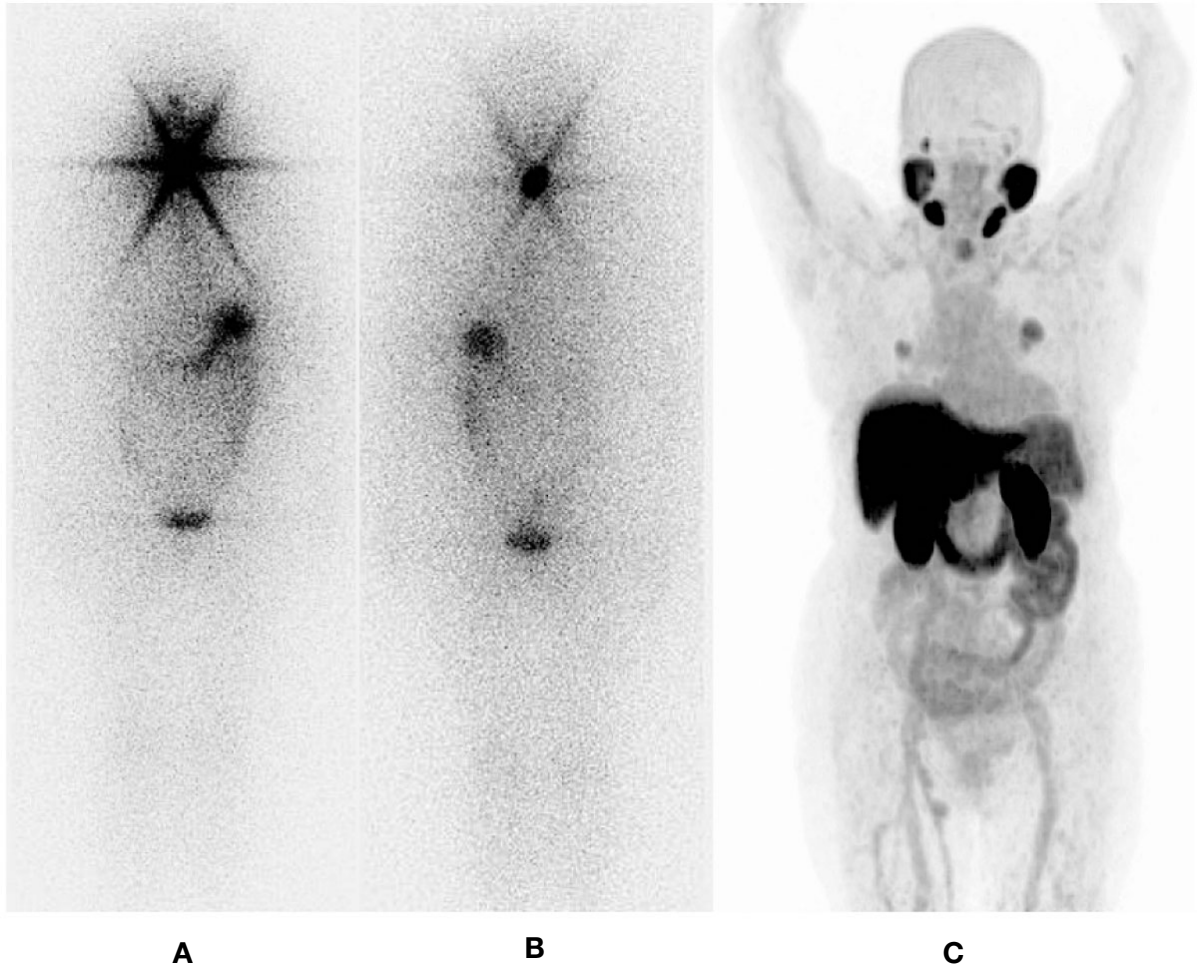
65 years old female; Thyroglobulin 253 ng/dl; treated with TT+RAI (14800 MBq). First two images show an ^131^I – post therapy whole body scan in **(A)** anterior and **(B)** posterior projections, with a star – shapped intense uptake. The image on the right **(C)**, shows ^68^Ga – PSMA PET/CT maximum intensity projection [MIP], which manifests a glaring superiority regarding spacial resolution, and target–to–background ratio.

**Figure 9:**
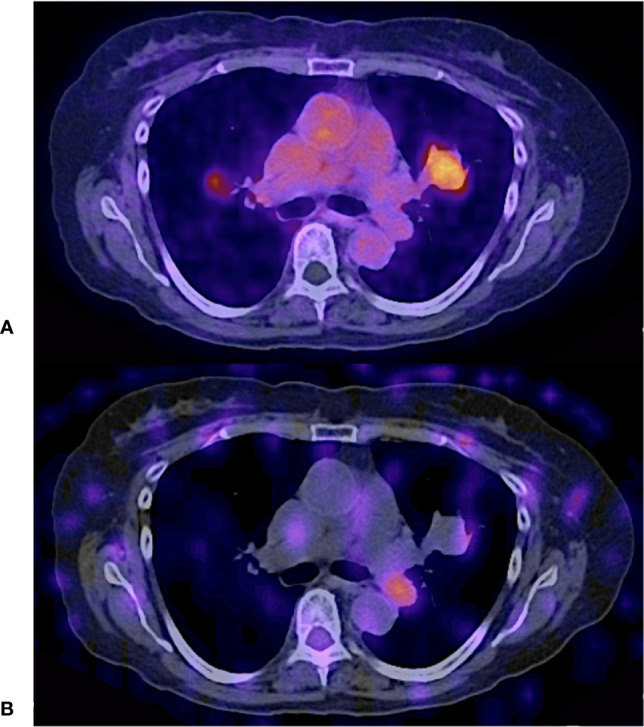
65 years old female; Thyroglobulin 253 ng/dl; treated with TT+RAI (cumulative dose 14800 MBq) **(A)** ^68^Ga - PSMA PET/CT **(B)** ^131^I – SPECT/CT.

**Figure 10:**
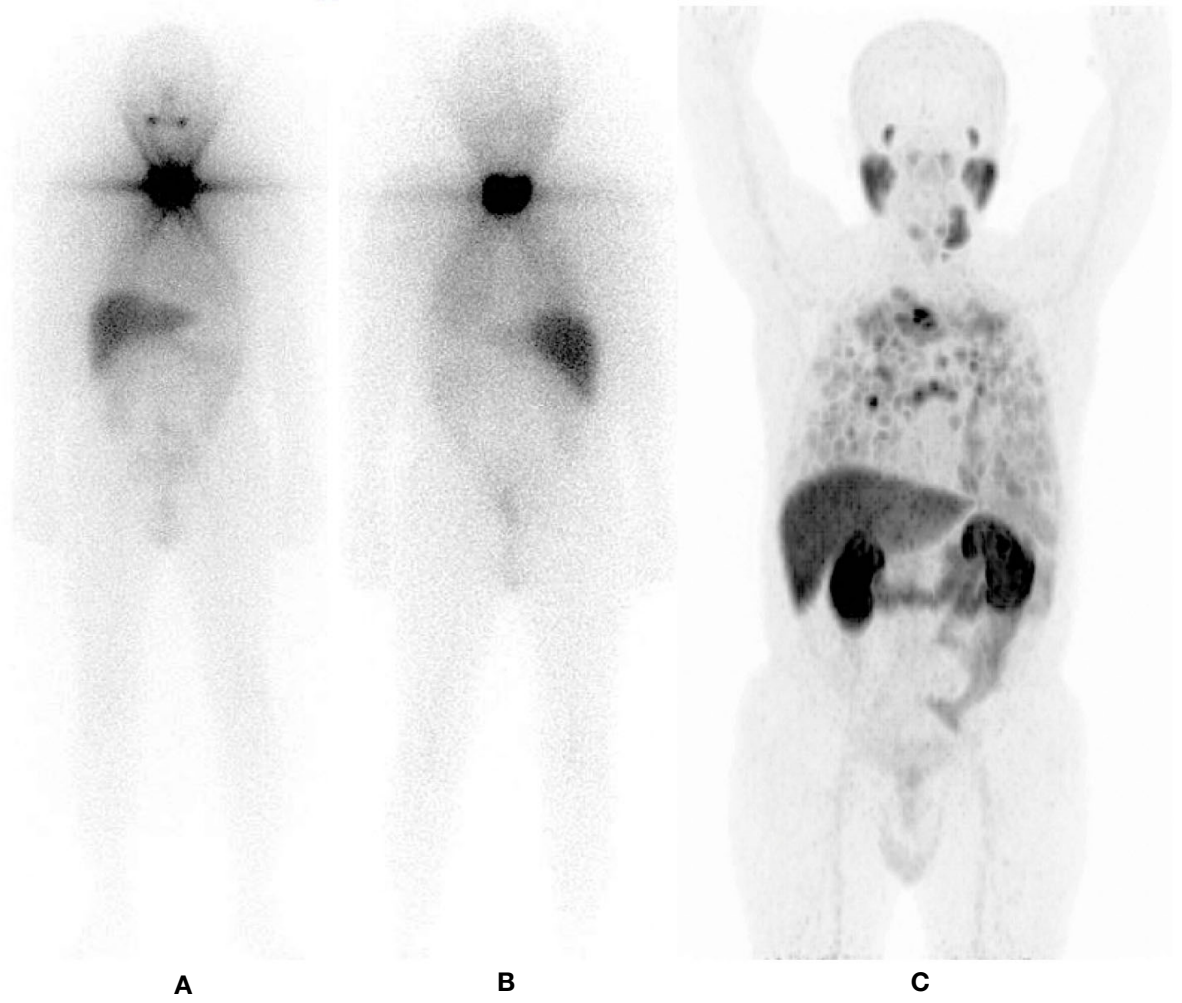
64 years old male, papillary thyroid carcinoma classic variant; treated with TT+RAI (cumulative dose 7400 MBq); Thyroglobulin 264.5 ng/dl. **(A, B)** ^131^I – post therapy whole body scan shows a focal uptake in thyroid bed and difusse concentration in lungs. **(C)** ^68^Ga – PSMA PET/CT maximum intensity projection with multiple lung and lymph node lesions PSMA avid.

**Figure 11:**
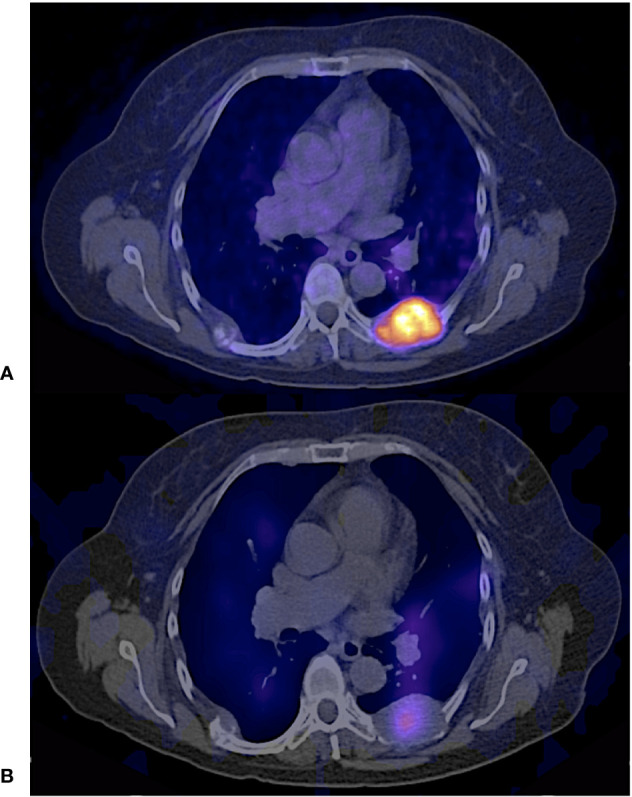
61 years old female with follicular thyroid carcinoma, insular differentiation (20%), treated with TT + RAI (cumulative dose 25900 MBq); Thyroglobulin 3922 ng/dl. Paravertebral implant. **(A)** ^68^Ga - PSMA PET/CT avid lesion. **(B)** ^131^I – SPECT/CT showed diffuse uptake of the radiotracer.

64/64 of lesions (100%) fulfilled ^68^Ga PSMA – 11 PET/CT positivity criteria. The main image criterion of positivity was the presence of focal uptake areas in one or more locations and/or higher than in the surrounding tissue background regardless of the presence or absence of lesion in the corresponding CT images. whereas ^131^I SPECT/CT detected 55/64 lesions (85.9%). Discrepant lesions (n = 9) were localized in lung 44.4% (n = 4) (**Figure 1**), brain 22.2% (n = 2), postoperative thyroid bed 11.1% (n = 1), lymph nodes 11.1% (n = 1) and bone 11.1% (n = 1) (**Figure 2**). In PET/CT median values resulted as follows: SUVmax 7.25, SD 11.8, (range from 1.8 to 70.5), TBR 5.8, SD 6 (1.8 – 35). While in SPECT/CT median value of counts were 40, SD 4,364.6 (0 – 35,000), TBR 6.1 and SD 114.8 (0 – 714). Higher uptake of PSMA was seen in brain metastases (**Figures 3**–**5**), crosswise it showed a lower uptake in malignant lymph nodes (**Figures 6**, **7**). In contradistinction, greater uptake was seen with ^131^I imaging in residual thyroid tissue (**Figure 8**), whilst diminished uptake was observed in lung lesions with this radiotracer (**Figure 9**). Although median TBR was greater in ^131^I SPECT/CT, SD was also higher; this was since in patient number 1, residual thyroid tissue was identified, which had substantial radioiodine avidity, causing an enormous amount of scattering data (**Figure 10**). In the rest of the patients, TBR from PET/CT was greater, meaning they had a much better target–to–background ratio (**Figure 11**). The degree of correspondence among observers was outstanding for both radiotracers, but close upon perfect for PSMA – 11 (κ = 0.98; 95% CI, 0.80 – 0.91), as opposed to ^131^ I (κ = 0.86; 95% CI, 0.71 – 0.76).

## Discussion

This study describes to the best of our knowledge, the first experience regarding ^68^Ga-PSMA PET/CT vs ^131^I SPECT/CT imaging in patients with well – differentiated metastatic thyroid carcinoma. PSMA PET/CT detected various types of lesions including central nervous system, lymph nodes, pulmonary nodules, and bone metastases. These findings are similar to those of Verma et al. and Lütje et al., who also detected these and various other lesions (16, 17). PSMA expression is frequently observed in the tumor-associated neovasculature of multiple neoplasms, including thyroid cancer. A systematic review by Bertagna et al. on thyroid incidentalomas with ^68^Ga – PSMA, showed that up to 23% of the detected lesions, corresponded to malignant – type lesions (18). There are other reviews that demonstrate a high expression of PSMA receptors in the microvasculature of thyroid neoplastic cells, which has risen the feasibility to evaluate this transmembrane protein; nevertheless, some studies carried out in microarrays have shown a variable uptake in well differentiated histologies. Apparently, the expression of PSMA is related to size and vascular invasion in follicular histologies (19, 20). Moore et al., demonstrated the immunohistochemical PSMA expression in benign and malignant thyroid tissue, as well as in metastatic tissue and infiltrated lymphatic nodes, the first of them, resulting in a higher degree of the antigen expression. It has also been observed that PSMA expression depends on histology; moderate to high grade expression of PSMA has been documented in classic papillary, follicular and papillary with follicular variant histologies, as well as in radioiodine – refractory subtypes (RAIR); and weak to null expression in anaplastic thyroid carcinoma (21). PSMA is also implicated in the generation of glutamate *via* its enzymatic action on N–acetyl–aspartyl–glutamate (NAAG). This transmembrane protein is required for liberation of glutamate from tumor – derived NAAG, although this relationship has not yet been established in the context of PCa. It has been shown that PSMA generates a localized reservoir of glutamate from NAAG and activates tumor growth in some neoplastic cells, like in high – grade ovarian serous adenocarcinoma (22). It is described that PSMA uptake may have some false positives related to inflammatory processes and non-prostatic malignancies such as clear cell renal carcinoma, hepatocarcinoma, among others (23). In the case of our study, all of the lesions were correlated with tomographic findings.

Our results demonstrate that PSMA detected more metastatic lesions than ^131^I, which could be related to the expression of type II carboxypeptidase in the vascular endothelium (19). All the lesions manifested PSMA uptake regardless of their iodine avidity; despite it, there was a single patient who received an ^131^I ablation dose, in whom postoperative thyroid bed showed more radioiodine avidity. The pathology report revealed negative post-surgical margins, therefore, the intense uptake of iodine, corresponds to the presence of non-tumoral thyroid tissue, which is why it did not exhibit PSMA avidity. This displays congruity with the information published in *The Atlas of Human Proteins*, where it is observed both at RNA and proteomic levels, that benign thyroid tissue has no expression of FOLH1 (24, 25).

We recognize that patients involved in this study do not have homogenous characteristics, which certainly represents a weakness in our study. As it is a pilot retrospective study, we only evaluated molecular phenotype characteristics of PSMA uptake. Also, one of the main problems regarding SPECT/CT in patients with well differentiated thyroid cancer and their follow-up, is spatial resolution, and that there are currently some other iodine radioisotopes that allow PET/CT realization, such as ^124^I, which would also permit a better head–to–head comparison; however, there is very few availability worldwide and it is also quite expensive. Although both radiotracers studies had 6 weeks interval, progression is not likely, since patients had just received an ^131^I therapeutic dose. Furthermore, there is evidence that sustains mean time-to-progression is 2 years (26).

Although the few numbers of patients evaluated was a limitation, this was compensated by the significant number of lesions that were observed in the study. These findings indicate the potential clinical usefulness of ^68^Ga – PSMA PET/CT; not only because it is able to depict tumor lesions in various locations, but also because it may detect lesions which are not visualized by ^131^I SPECT/CT. Furthermore, ^68^Ga – PSMA PET/CT could be used for theragnostic application in selected patients for possible therapy with ^177^Lu – PSMA–617. Vries et al., described the experience of employing ^68^Ga – PSMA PET/CT imaging and subsequent therapy of metastatic RAIR DTC using ^177^Lu – PSMA–617, where one of the two patients who underwent radioligand therapy, showed slight, temporary response (27). Literature is scarce regarding ^177^Lu – PSMA therapy in patients with RAIR DTC, Assadi and Ahmadzadehfar presented another case report in which both ^177^Lu – PSMA and ^177^Lu – DOTATATE was given to a patient (28). Since PSMA uptake mechanism does not depend on iodine – sodium (I – Na) symporters, TSH stimulation is not necessary for theragnostic purposes. This evidence suggests that PSMA could be an alternative for diagnosis and treatment in patients diagnosed with thyroid cancer; nonetheless, future prospective studies should be performed.

## Conclusions

In patients with well differentiated thyroid cancer, ^68^Ga – PSMA PET/CT detects a greater number of lesions than ^131^ I, acknowledging CT as reference. In none of the cases, were there lesions observed by radioiodine imaging, that were not detectable by CT. Since PSMA uptake mechanism is I – Na symporter independent, its application in patients with this type of cancer, may have several benefits and/or advantages over the actual SOC imaging modality, being the first of them not requiring levothyroxine suppression, as well as identifying precociously, lesions with a more aggressive potential that could be going through radioiodine refractoriness. Furthermore, taking into consideration the degree of PSMA uptake by metastatic lesions, theragnostic purposes ought to be considered in future prospective trials.

## Data Availability Statement

The raw data supporting the conclusions of this article will be made available by the authors, without undue reservation.

## Ethics Statement

The studies involving human participants were reviewed and approved by Comité de Etica del Instituto Nacional de Cancerología. Written informed consent for participation was not required for this study in accordance with the national legislation and the institutional requirements.

## Author Contributions

QP-C, JV-A, SG-R, and FG-P conceptualized the study. FG-P, QP-C, and LT-A analyzed and interpreted the data and wrote the manuscript. QP-C, JV-A, SG-R, IS-G, EG-A, LT-A, and FG-P participated in scientific discussions and revised the manuscript. All authors contributed to the article and approved the submitted version.

## Conflict of Interest

The authors declare that the research was conducted in the absence of any commercial or financial relationships that could be construed as a potential conflict of interest.

## Publisher’s Note

All claims expressed in this article are solely those of the authors and do not necessarily represent those of their affiliated organizations, or those of the publisher, the editors and the reviewers. Any product that may be evaluated in this article, or claim that may be made by its manufacturer, is not guaranteed or endorsed by the publisher.
